# Acute Moderate-Dose β-Alanine Improves Exercise Efficiency via Bicarbonate-Related Mechanisms During a Cycling Time Trial

**DOI:** 10.3390/sports14060252

**Published:** 2026-06-20

**Authors:** Juan Carlos Muñoz-Carrillo, Silvia Pérez-Piñero, Francisco Javier López-Román, Antonio J. Luque-Rubia, Vicente Ávila-Gandía

**Affiliations:** 1 Health Sciences Department, Universidad Católica San Antonio de Murcia UCAM, Campus de los Jerónimos n°135, Guadalupe, 30107 Murcia, Spain; jcmunoz@ucam.edu (J.C.M.-C.); jlroman@ucam.edu (F.J.L.-R.); ajluque@ucam.edu (A.J.L.-R.); vavila@ucam.edu (V.Á.-G.); 2 Health Sciences PhD Program, Universidad Católica de Murcia UCAM, Campus de los Jerónimos n°135, Guadalupe, 30107 Murcia, Spain; 3 Primary Care Research Group, Biomedical Research Institute of Murcia (IMIB-Arrixaca), 30120 Murcia, Spain

**Keywords:** high dose, hydrogen ions, performance, sustained-release formulation, pH

## Abstract

Background: Research on the acute effects of β-alanine supplementation has primarily focused on performance outcomes, with limited attention to the underlying physiological mechanisms. This study aimed to investigate the acute effects of two β-alanine doses on performance, mechanical output, and acid–base balance during a 10 min cycling time trial (10’-TT), and to explore the relationship between buffering-related variables and performance. Methods: Eighty-five recreational cyclists performed a 10’-TT under indoor conditions before (control) and following the acute ingestion of β-alanine (moderate-dose β-alanine 10 g—BAM; high-dose β-alanine 20 g—BAH) or placebo (PLA), with each condition tested on separate days. Data were analyzed using two-way repeated-measures ANOVA and correlation analyses. Results: No significant differences were observed in performance variables (distance, speed, cadence, or heart rate; *p* ≥ 0.751). However, total external mechanical work (kJ) was significantly reduced following acute supplementation (*p* = 0.028). Notably, the BAM condition reduced the mechanical cost of exercise without impairing performance, and this effect was moderately associated with changes in bicarbonate levels. Conclusions: Acute β-alanine supplementation did not improve performance outcomes but may alter buffering-related physiological responses associated with reduced mechanical work during high-intensity cycling exercise. These findings highlight the relevance of buffering-related mechanisms, particularly bicarbonate dynamics, in modulating the mechanical cost (work performed relative to performance achieved) of high-intensity exercise.

## 1. Introduction

High-intensity exercise performance is largely limited by the accumulation of metabolic by-products, particularly hydrogen ions (H^+^), which contribute to the development of metabolic acidosis and fatigue [1]. While early theories attributed fatigue primarily to lactate accumulation, current evidence indicates that disturbances in acid–base balance, ionic homeostasis, and excitation–contraction coupling are key determinants of muscular performance decline [2,3]. These alterations impair enzyme activity, reduce calcium sensitivity, and ultimately compromise force production during high-intensity efforts [3].

To counteract exercise-induced acidosis, both intracellular and extracellular buffering systems are activated. Intracellular buffering is primarily mediated by carnosine, a dipeptide synthesized from β-alanine and histidine, which plays a key role in maintaining intracellular pH [4]. In parallel, extracellular buffering depends largely on bicarbonate (HCO_3_^−^), which facilitates the efflux of H^+^ from muscle cells and contributes to systemic acid–base regulation. Together, these buffering systems are critical determinants of exercise tolerance, particularly in efforts lasting between 1 and 10 min [5,6].

β-alanine is a non-proteogenic amino acid and the rate-limiting precursor for carnosine synthesis in skeletal muscle. Therefore, β-alanine supplementation has been widely proposed as an effective nutritional strategy to enhance muscle buffering capacity and improve exercise performance [7,8]. Indeed, β-alanine is currently classified as a category A ergogenic aid, supported by a substantial body of evidence demonstrating its efficacy across a wide range of athletic populations [7,9,10,11].

The ergogenic effects of β-alanine are primarily attributed to its role in increasing intramuscular carnosine content, thereby enhancing intracellular buffering capacity. However, additional mechanisms have also been proposed, including improvements in oxidative metabolism during high-intensity exercise [12], increased calcium sensitivity at the level of actin–myosin interaction, and enhanced excitation–contraction coupling [13]. These adaptations may collectively contribute to improved force production and altered physiological responses during high-intensity exercise.

Traditional β-alanine supplementation protocols aimed at increasing muscle carnosine content typically involve daily doses ranging from 4.6 to 6.4 g over a period of 4 to 10 weeks, resulting in cumulative intakes of approximately 180–250 g [10,14,15]. However, larger cumulative doses have been shown to induce greater increases in muscle carnosine, with reports indicating up to a 50% increase following total intakes of ~230 g [16,17,18]. More recently, dose–response modelling studies have suggested that a cumulative intake of approximately 377 g is required to achieve 50% of maximal carnosine saturation, while intakes approaching 1000 g may be necessary to achieve ≥70% of the maximal response [19].

Despite the well-established effects of chronic β-alanine supplementation, these traditional protocols require an extensive timeframe (4–10 weeks) to induce performance benefits, which may present practical limitations regarding athlete adherence and immediate competition needs. Consequently, there is a compelling rationale to search for more efficient alternatives, particularly in terms of time. From a practical perspective, acute supplementation strategies may represent a more time-efficient alternative to traditional chronic loading protocols. However, considerably less is known about its acute effects. When β-alanine is administered as a single dose, the ergogenic outcomes appear to be inconsistent, possibly due to the relatively small doses used in most studies. For instance, Barahona et al. [20] reported improvements in time-to-exhaustion following acute β-alanine ingestion in middle-distance runners, whereas Glen et al. [21] found no improvements in Wingate performance in moderately trained cyclists following ingestion of 2.4 g of β-alanine. In this line, Invernizzi et al. [22] reported no improvements in RPE, blood lactate concentration, glucose and HCO_3_^−^, on anaerobic intermittent performance in young active participants after acute ingestion of 1 g of β-alanine.

One of the key limitations of previous studies is the relatively small acute dose administered, which may be insufficient to induce detectable physiological changes. Therefore, the potential ergogenic effects of larger acute doses of β-alanine remain largely unexplored. Investigating whether an acute, high-dose strategy could represent a viable, time-efficient alternative to chronic protocols is therefore warranted.

In recent years, emerging evidence has begun to explore the effects of larger acute doses of β-alanine (12–20 g), particularly using sustained-release formulations designed to improve tolerability. For instance, Pérez-Piñero et al. [23] reported that the ingestion of four 5 g doses during one week of sustained-release β-alanine improved relative mean power output during an uphill time-trial in professional World Tour cyclists. Similarly, Ávila-Gandía [24] observed an attenuation of performance decrements during a 10 min time trial (10’-TT) following intensive training camp in the same population.

One of the main limitations associated with large-dose β-alanine supplementation is the occurrence of paresthesia, a side effect characterized by tingling or itching sensations, which are typically reported with single doses exceeding 1.6 g or 10–20 mg/kg of body mass, and is thought to be related to rapid increases in plasma β-alanine concentration [25]. To mitigate these effects, sustained-release formulations have been developed, allowing for larger doses to be administered without inducing paresthesia, thereby enabling the investigation of large-dose acute supplementation strategies [26].

In addition to maximal performance output, the relationship between external workload and physiological demand represents an important determinant of endurance performance [27,28]. However, the potential role of acute β-alanine supplementation in modulating buffering-related responses associated with mechanical demand during exercise has not been previously investigated.

Furthermore, research on the acute effects of β-alanine supplementation has largely been limited to performance outcomes, with minimal exploration of the underlying biochemical mechanisms. This represents a critical gap in the literature, as performance changes cannot be fully interpreted without understanding the physiological processes that drive them. While pH is commonly used as a marker of acidosis, it may lack sensitivity to detect subtle alterations in buffering capacity. In contrast, HCO_3_^−^ plays a central role in extracellular buffering and may provide a more mechanistically relevant indicator of acid–base regulation. Therefore, investigating bicarbonate dynamics in relation to mechanical output may yield novel insights into the physiological basis of performance changes following acute β-alanine ingestion.

Therefore, the aim of the present study was to investigate the acute effects of two different large doses of β-alanine supplementation (10 g and 20 g) on performance, mechanical output, and acid–base balance during a 10’-TT. In addition, this study sought to explore the relationship between changes in buffering-related variables and performance outcomes, with a particular focus on the potential mechanistic role of bicarbonate dynamics in modulating the mechanical cost of exercise.

## 2. Materials and Methods

### 2.1. Study Design

A randomized, controlled, double-blind, single-center clinical trial with three parallel arms was conducted based on the product consumed and dosage: moderate-dose β-alanine (BAM), high-dose β-alanine (BAH), and placebo (PLA). Participants visited the laboratory on three separate occasions, each spaced 7 days apart. To analyze the acute effect of β-alanine intake on cycling performance, a 10’-TT was conducted under indoor conditions, first without product consumption (control). Then, after 7 days, a second 10’-TT was conducted with β-alanine or PLA intake (acute effects). Participants were stratified according to the maximal oxygen consumption obtained during the cardiorespiratory fitness test [29] and then randomly allocated to one of the three experimental groups. The randomization process was conducted by a researcher who was not involved in the study’s implementation or evaluation. For this purpose, a software program (Epidat 4.2, Galicia, Spain) generated randomized codes, which were then assigned to participants. The study protocol and informed consent form were approved by the Ethics Committee of San Antonio Catholic University (code CE092104) (Murcia, Spain) and registered at ClinicalTrials.gov (NCT06180512). The study was conducted in accordance with the Declaration of Helsinki and followed the CONSORT guidelines for randomized controlled trials (RCTs). Written informed consent was obtained from all participants.

### 2.2. Participants

Participants were recruited from the clinical studies database available at Universidad Católica San Antonio de Murcia. A total of eighty-five recreational cyclists (age = 36.4 ± 12.3 years, body mass = 73.1 ± 8.6 kg, height = 175.3 ± 6.1 cm) were included. Inclusion criteria included (a) Male cyclists training at least twice a week (a minimum of 4 h per week) with more than two years of cycling experience; (b) Ability to perform tests without signs of fatigue. The exclusion criteria were: (a) Presence of chronic diseases; (b) Current or past injuries that prevented normal training in the month prior to the study; (c) Allergy to β-alanine, wheat, or any of the placebo ingredients (wheat semolina); (d) β-Alanine intake within the two months prior to the study; (e) Use of any type of ergogenic aid that could enhance performance within the last 15 days; (f) Inability or refusal to understand and sign the informed consent before participation. The research was carried out in January 2024. Sample size estimation was based on mean power output (MPO) during the 10’-TT. Considering the effects reported by Bellinger and Minahan [30], an alpha level of 0.05, a statistical power of 80%, and an expected precision of 10 W, a minimum of 28 participants per group was considered appropriate for the repeated-measures design.

### 2.3. Product and Supplementation Protocol

Cyclists were randomly assigned to one of three groups: BAH (20 g of sustained-release β-alanine microgranule powder blend), BAM (10 g of sustained-release β-alanine microgranule powder blend) (BETA-FOR3MAX^®^, Martínez Nieto S.A., Cartagena, Spain), or PLA group (uncooked wheat semolina from Triticum durum). The selected doses were based on previous studies using the same sustained-release formulation, which demonstrated good tolerability and absence of severe adverse effects under high acute dosing conditions [23,24]. The placebo had similar organoleptic characteristics and no ergogenic effects. The placebo was selected to match the organoleptic characteristics of the supplement. Both β-alanine and PLA were produced by the same manufacturer and were provided in opaque, sealed containers that were weighed to ensure consistency and labeled for proper identification. A researcher not involved in the study was responsible for preparing the containers with the precise amounts using a precision scale.

During the third visit (acute effects refers to a single-day protocol, in which β-alanine was administered in multiple doses within the same day prior to performance assessment), participants in the BAM group ingested four doses of 6.54 g of product (equivalent to 2.5 g of pure β-alanine per dose) at 75 min intervals. Two hours elapsed between the final intake and the start of the warm-up. The BAH group followed the same protocol but consumed four doses of 13.09 g of product (5 g of pure β-alanine per dose). The PLA group carried out the same protocol as the other groups and ingested four doses of 6.54 g of placebo.

The total accumulated product intake was 26.16 g for the BAM group and 52.36 g for the BAH group (including encapsulation coating + 10 g or 20 g of pure β-alanine, respectively). The PLA group consumed a total of 26.16 g of uncooked wheat semolina. According to De Salazar et al. [26], the administration of large daily doses of β-alanine appears to be an effective short-term strategy to enhance systemic availability. Participants were required to consume the entire contents of each container. All groups received the same number of opaque containers to ensure blinding of the study. They were instructed to use water as needed to ensure complete intake.

Compliance with supplementation was assessed by comparing the total amount of product provided at baseline with the amount returned at the end of the study. Only participants who consumed at least 80% of the total prescribed dose were included in the analysis.

Participants were instructed to maintain their normal dietary intake habits throughout the investigation. Participants were required to record all food intake 72 h before their visits at control 10’-TT and acute effects 10’-TT.

Paresthesia was assessed using a subjective evaluation questionnaire administered at two time points: baseline (control) and prior to the 10’-TT following acute supplementation, in accordance with the procedure described by Ávila-Gandía et al. [24]. The questionnaire included both quantitative (intensity) and qualitative (type of sensation) items.

### 2.4. Intervention and Study Procedures

Participants carried out three testing sessions, separated by 2 weeks. During the first session, participants performed a maximal incremental ramp test (MIRT) with their personal road bicycles mounted on a Cyclus 2 ergometer (electronik-automation GmbH, Leipzig, Germany), which has been demonstrated to be validated [31]. The results of this test were used to determine maximal oxygen uptake (VO_2_max) and to stratify participants according to their performance level. Additionally, the MPO attained at VO_2_max was used to standardize the warm-up protocol for the 10’-TT. The protocol consisted of a 3 min self-paced warm-up, followed by a MIRT starting at 50 watts (W), with increments of 5 W every 12 s (equivalent to 25 W/min). Participants pedaled at a self-selected cadence between 60–100 revolutions per minute (RPM) using a fixed gear ratio, chosen prior to the start of the test.

#### Ten-Minute Cycling Time Trial (10’-TT)

During the second and third visits, participants performed a 10’-TT under two conditions: without product intake (control condition) and following acute supplementation with BAM, BAH, or PLA (acute effects condition). All the 10’-TTs were conducted during the same evening hours (between 15:00 PM and 19:00 PM) under controlled environmental conditions (temperature: 20–22 °C; relative humidity: 40–60%). Each cyclist used their own personal road bike mounted on a Tacx Neo 2T Smart Trainer (Tacx International, Rijksstraatweg, The Netherlands) [32]. Before each 10’-TT, participants completed a standardized warm-up consisting of 10 min at 50% of the maximum power output reached during the MIRT, including three short sprints performed at minutes 5, 7, and 9, followed by 2 min of active recovery. Participants were instructed to cover the greatest possible distance within the 10 min time limit using a self-paced strategy. To minimize external feedback and reduce performance bias, all performance variables displayed on the Garmin Edge 530 cycle computer (Garmin Ltd., Olathe, KS, USA), including power output, cadence, heart rate, speed, and distance, were hidden from participants, except for elapsed time. The following performance variables were recorded during the 10’-TT: total distance covered, total mechanical work, mean power output, relative power output, cadence, speed, and heart rate. Upon completion of the test, participants engaged in 3 min of active recovery at a self-selected cadence and power output. All performance data were uploaded to the Garmin Connect™ platform (https://connect.garmin.com/es/, accessed in 1 January 2024) and subsequently exported for analysis using the Golden Cheetah training software (version 3.5, https://connect.garmin.com/es/, accessed in 1 January 2024).

Heart rate during the 10’-TT was continuously monitored using a Garmin HRM-Dual chest strap (Garmin Ltd., Olathe, KS, USA) [33]. The rate of perceived exertion (RPE) was assessed using a modified 10-point Borg Scale [34], recorded before and after the warm-up and immediately post-exercise. Physiological and perceptual responses were also monitored throughout the protocol.

Additionally, microcapillary blood samples (65 μL) were collected at baseline (1 min before starting the warm-up, basal), at the end of the warm-up (after 2 min of active recovery, Pre 10’-TT), and immediately after the 10’-TT (Post 10’-TT). These samples were analyzed using a blood gas analyzer (ABL90 FLEX, Radiometer Medical APS, Copenhagen, Denmark), which provided several physiological and acid–base variables, including blood gas parameters (tCO_2_ and CO_2_), excess standard base (SBE), HCO_3_^−^, pH, anion gap, metabolic variable (lactate) [35].

### 2.5. Bias Control Variables

To minimize potential sources of bias, several control procedures were implemented throughout the study. Participants were instructed to maintain their usual training habits and to avoid strenuous exercise, alcohol, and caffeine intake for 24 h prior to each testing session. Body mass was monitored before each trial to ensure stable hydration and nutritional status. Participants completed a 72 h dietary record before both the control and acute testing sessions. Dietary records were analyzed to estimate total energy intake, macronutrient distribution, and dietary histidine intake. These variables were used to verify dietary standardization between testing sessions and across groups. In addition, all tests were performed under standardized environmental conditions and at the same time of day for each participant whenever possible.

### 2.6. Statistical Analysis

All variables were analyzed using a two-way repeated-measures ANOVA with group (PLA, BAM, BAH) as the between-subject factor and time (PRE vs. POST) as the within-subject factor. When appropriate, post hoc analyses were performed. Prior to statistical analyses, data normality was assessed using the Shapiro–Wilk test and visual inspection of Q–Q plots. Repeated-measures ANOVA and Pearson correlation analyses were considered appropriate due to the robustness of these methods to moderate deviations from normality and the balanced sample sizes across groups.

Individual changes (Δ) were calculated for all variables, and additional normalized mechanical variables (e.g., work relative to distance covered) were derived. Pearson correlation analyses were conducted to examine the relationships between changes in mechanical variables and acid–base parameters within each group. To interpret the practical relevance and strength of the correlation coefficients (*r*), the scale proposed by Hopkins et al. (2009) [36] was adopted, where |*r*| < 0.10 was considered trivial, 0.10 ≤ |*r*| ≤ 0.29 small, 0.30 ≤ |*r*| ≤ 0.49 moderate, 0.50 ≤ |*r*| ≤ 0.69 large, 0.70 ≤ |*r*| ≤ 0.89 very large and |*r*| ≥ 0.90 nearly perfect. Additionally, the coefficient of determination (*r*^2^) was calculated for each correlation to estimate the proportion of shared variance, allowing a clearer distinction between statistical significance and potential practical relevance.

Statistical significance was set at *p* ≤ 0.05. All analyses were performed using SPSS (version 25.0; SPSS Inc., Chicago, IL, USA).

## 3. Results

The flow of participants throughout the study is presented in the Supplementary Material (Figure S1). Eighty-five participants were randomized to PLA (*n* = 28), BAM (*n* = 28), or BAH (*n* = 29). A total of 75 participants completed the study and were included in the final analyses (PLA: *n* = 22; BAM: *n* = 26; BAH: *n* = 27), with no exclusions at the analysis stage.

### 3.1. Participant Characteristics

Baseline descriptive characteristics are presented in Table 1. No significant differences were observed between groups for age, anthropometric variables, body composition, VO_2_max, indicating adequate baseline homogeneity across experimental conditions.

No significant differences were observed between groups for total energy intake, protein, carbohydrate, fat, or histidine intake (all *p* > 0.05). Similarly, no significant between-group differences were found for relative protein and histidine intake (Table 2).

Paresthesia-related symptoms were generally mild across all conditions. No symptoms were reported during the control condition. Following acute supplementation, the mean paresthesia score remained low in all groups (PLA: ~1.9 ± 2.4; BAM: ~2.5 ± 2.4; BAH: ~2.6 ± 2.5), with no significant differences observed between groups (*p* = 0.570). General tingling was the most frequently reported sensation, particularly in the BAH condition, where a greater frequency and variety of symptoms were observed. No participant discontinued the protocol due to adverse effects.

### 3.2. Performance Outcomes During the 10’-TT

Performance variables obtained during the 10’-TT are presented in Table 3. No significant effects were observed for total distance covered (PLA: *p* = 0.382; BAM: *p* = 0.394; BAH: *p* = 0.521), average speed (*p* ≥ 0.629), cadence (*p* ≥ 0.150), or heart rate (*p* ≥ 0.751). However, significant effects were observed for variables related to mechanical output.

Total work showed a significant reduction following acute supplementation (*p* = 0.028). This effect was driven by the BAM group, which exhibited a significant decrease (*p* = 0.004), whereas no significant changes were observed in the PLA (*p* = 0.346) or BAH groups (*p* = 0.406). Similarly, mean power output was significantly reduced (*p* = 0.038), with a decrease observed in the BAM group (*p* = 0.011), while no differences were found in PLA (*p* = 0.255) or BAH (*p* = 0.453). Relative power also showed a significant effect (*p* = 0.046), with a reduction in the BAM group (*p* = 0.047), whereas no significant changes were observed in PLA (*p* = 0.119) or BAH (*p* = 0.632).

### 3.3. Ratings of Perceived Exertion (RPE)

RPE values at baseline, post-warm-up, and at the end of the time trial are presented in Table 4. No significant differences were observed between groups at baseline prior to supplementation (*p* = 0.581). Following the warm-up, a significant reduction in RPE was observed in the BAM group after acute supplementation (*p* = 0.050), whereas no changes were detected in placebo or BAH. However, no significant differences between-groups were found (*p* = 0.466). At the end of 10’-TT, no significant differences were observed between conditions or groups (*p* = 0.304).

### 3.4. Blood Biomarkers and Acid–Base Balance

Blood acid–base and metabolic responses are presented in the Supplementary Material (Table S1). No significant differences were observed at baseline or pre-exercise for any variable (*p* > 0.05).

**Figure 1:**
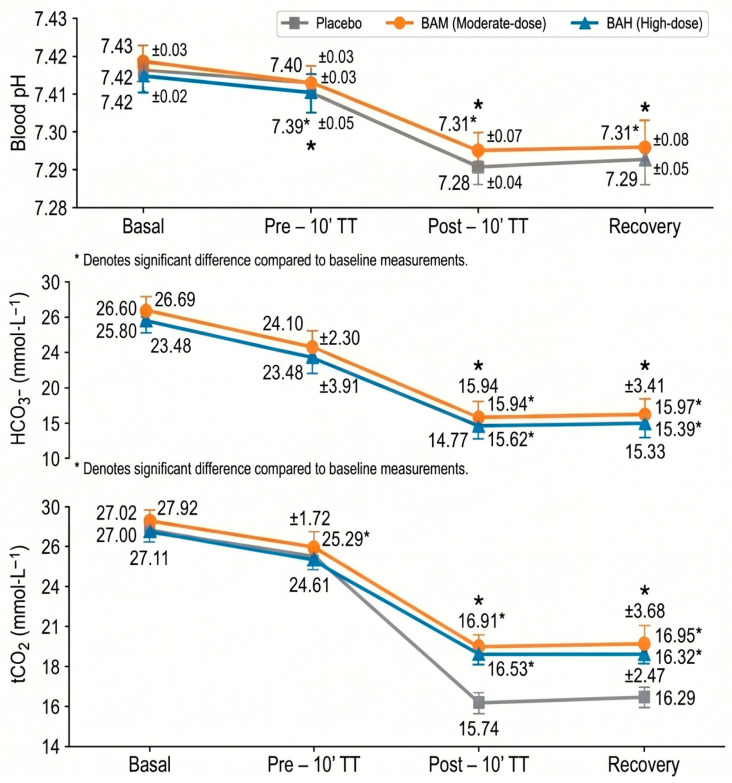
Physiological impact of acute β-alanine supplementation on blood acid–base responses during a high-intensity cycling time trial. Changes in blood pH, bicarbonate (HCO_3_^−^), and total carbon dioxide (tCO_2_) measured at baseline, pre-exercise, immediately post 10 min time trial (10’-TT), and recovery following acute supplementation with placebo (PLA), moderate-dose β-alanine (BAM; 10 g), or high-dose β-alanine (BAH; 20 g). Values are presented as mean ± SD. Both β-alanine conditions showed attenuated reductions in bicarbonate and tCO_2_ following exercise compared with placebo, suggesting a potential modulation of extracellular buffering responses. Significant differences from baseline within each group are indicated by * (*p* < 0.05).

A significant condition effect was observed for blood pH at post-10’-TT (*p* = 0.018) and after 3 min of recovery (*p* = 0.024). Specifically, higher pH values were observed following acute supplementation in the BAM group at both time points (post-10’-TT: 7.31 ± 0.07 vs. 7.28 ± 0.04; recovery: 7.31 ± 0.08 vs. 7.29 ± 0.05). Similarly, HCO_3_^−^ concentration differed significantly at post-10’-TT (*p* = 0.050) and recovery (*p* = 0.010), with greater values observed in both BAM and BAH compared to PLA (e.g., post-10’-TT: BAM = 15.94 ± 2.97 mmol·L^−1^, BAH = 15.62 ± 2.63 mmol·L^−1^, PLA = 14.77 ± 2.73 mmol·L^−1^) (Figure 1).

In addition, total CO_2_ (tCO_2_) was significantly increased at post-10’-TT (*p* = 0.049) and recovery (*p* = 0.008), particularly in the BAM group. SBE also differed significantly at post-10’-TT (*p* = 0.036) and recovery (*p* = 0.011), with less negative values following supplementation (BAH and BAM) (Figure 1).

No significant differences were observed for CO_2_, anion gap, or lactate concentrations at any time point (all *p* > 0.05). Lactate increased markedly after the 10’-TT in all groups, confirming the high metabolic demand of the protocol (baseline (~1.5–1.7 mmol·L^−1^) to post-10’-TT (~11.9–13.4 mmol·L^−1^)); however, no significant between-group or condition effects were observed (all *p* > 0.05). Full lactate values are reported in Supplementary Table S1.

### 3.5. The Relationship Between Performance and Biochemistry

#### 3.5.1. BAH Group and BAM Group

No significant correlations were observed between performance-related variables and acid–base parameters in the BAH group (all *p* > 0.40), suggesting the absence of a clear association between buffering capacity and mechanical output at this dose (Table 5).

**Figure 2:**
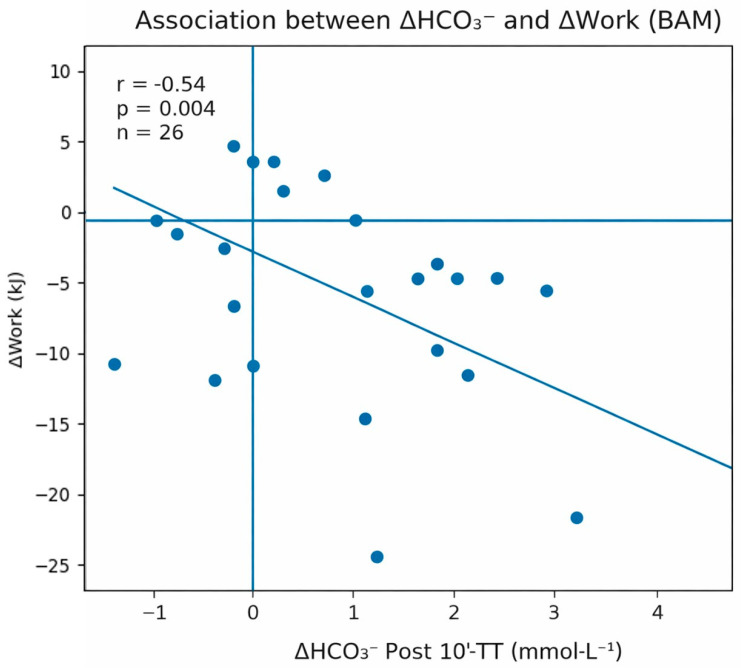
Association between the change in bicarbonate concentration immediately after the time trial (ΔHCO_3_^−^ Post-10’-TT) and the change in total mechanical work (ΔWork) in the BAM group. Each point represents an individual participant (*n* = 26). A significant negative correlation was observed (*r* = −0.54, *p* = 0.004), indicating that individuals exhibiting smaller reductions in bicarbonate concentration tended to require lower mechanical work during the 10’-TT.

In the BAM group, a large and statistically significant negative correlation was observed between changes in total mechanical work (ΔWork) and bicarbonate concentration immediately post-exercise (ΔHCO_3_^−^ Post-10’-TT) (*r* = −0.54, *p* = 0.004, *r*^2^ = 0.30). This association indicates that participants exhibiting smaller reductions in bicarbonate concentration tended to show larger reductions in total mechanical work (Figure 2). Notably, this association accounted for approximately 30% of the shared variance between variables

Regarding the remaining variables for the BAM and BAH groups, no other significant associations were found between performance variables and pH (*p* > 0.69, *r*^2^ ≤ 0.01), nor between efficiency-related variables (ΔWorkPerKm) and recovery bicarbonate levels in the BAM group (*r* = −0.28, *p* = 0.163, *r*^2^ = 0.08), representing a small effect size with limited shared variance (Table 5).

#### 3.5.2. PLA Group

In the PLA group, a moderate and statistically significant negative correlation was observed between changes in distance and pH (ΔDistance vs. ΔpH; *r* = −0.40, *p* < 0.05, *r*^2^ = 0.16), indicating that participants experiencing greater reductions in pH tended to cover shorter distances during the 10’-TT. Approximately 16.1% of the variance was shared between both variables.

Additionally, a moderate but non-significant negative trend was observed between changes in total work and both pH (ΔWork vs. ΔpH_Post 10’-TT; *r* = −0.31, *p* = 0.165, *r*^2^ = 0.09) and bicarbonate concentration (ΔWork vs. ΔHCO_3_^−^ Post 10’-TT; *r* = −0.31, *p* = 0.163, *r*^2^ = 0.10). Although these trends suggest a potential association between acid–base alterations and mechanical output, they did not reach statistical significance and explained 10% or less of the shared variance. No significant associations were found for the remaining variables (all *p* > 0.786, *r*^2^ ≤ 0.004) (Table 5).

## 4. Discussion

Despite the widespread use of β-alanine as an ergogenic aid, most previous research has focused on chronic supplementation protocols aimed at increasing intramuscular carnosine content. In contrast, the physiological and performance-related effects of acute high-dose β-alanine supplementation remain poorly understood, particularly regarding the underlying buffering-related responses during high-intensity exercise. From an applied perspective, understanding whether acute supplementation strategies may influence performance determinants could be relevant for athletes seeking shorter-term nutritional interventions when prolonged loading protocols are not feasible. The present study provides new insights into the acute effects of β-alanine supplementation on performance and physiological responses during a high-intensity cycling time trial. The main findings indicate that acute ingestion of β-alanine at a moderate dose (BA-10g) was associated with a reduction in total mechanical work without changes in performance outcomes. In addition, a significant association was observed between changes in mechanical work and bicarbonate concentration, whereas no such relationship was found with pH. Notably, these patterns were not evident in either the larger-dose condition (BA-20g) or the placebo group, suggesting a dose-specific and potentially non-linear response [7].

One relevant finding of this study was the dissociation between external performance (distance covered) and internal workload (mechanical work and power output) observed in the BAM condition. This pattern may suggest that a similar performance outcome was achieved with a lower mechanical demand during exercise. However, it should be acknowledged that efficiency was not directly measured using metabolic variables (e.g., oxygen consumption or energy expenditure), and therefore this interpretation should be considered with caution [27,28].

While β-alanine supplementation has been extensively studied in the context of chronic loading protocols aimed at increasing muscle carnosine content [16,18], considerably less is known about its acute effects, particularly when larger doses are administered within a single day. The present findings contribute to this emerging area by suggesting that acute large-dose β-alanine ingestion may influence performance-related outcomes through mechanisms that do not rely on long-term adaptations.

A key observation of the present study was the identification of a large and significant association between changes in bicarbonate concentration and mechanical work in the BAM condition, which accounted for 30% of the shared variance (*r*^2^ = 0.30). Bicarbonate plays a central role in extracellular buffering, facilitating the removal of H^+^ from the muscle and helping to maintain intracellular pH during high-intensity exercise [2]. Given the central role of bicarbonate in regulating acid–base balance, this finding could indicate that a smaller decrease in bicarbonate concentration is associated with reduced mechanical demands during exercise, without compromising performance. However, this interpretation should be considered cautiously, as the study design does not allow for direct mechanistic conclusions.

Interestingly, no association was found between pH and performance variables in the β-alanine conditions, whereas a significant relationship between pH and performance (distance) was observed in the PLA group. This finding is consistent with the well-established role of exercise-induced acidosis as a limiting factor in performance. In contrast, the absence of this relationship following β-alanine supplementation, together with the observed association with bicarbonate, may indicate that buffering dynamics—particularly bicarbonate availability—could play a more relevant role than pH per se under these conditions.

These findings align with previous literature highlighting the importance of buffering capacity, both intracellular (carnosine) and extracellular (bicarbonate), in high-intensity exercise performance [5,12,37]. In this context, the present results provide additional evidence suggesting that acute β-alanine supplementation may influence extracellular buffering-related responses, although further mechanistic studies are required to confirm this hypothesis.

Traditionally, the ergogenic effects of β-alanine have been attributed to increased intramuscular carnosine content, which enhances intracellular buffering capacity [4,25]. However, given the acute nature of the intervention in the present study, it is unlikely that significant increases in muscle carnosine occurred. Therefore, alternative mechanisms must be involved. It has been suggested that β-alanine could influence calcium sensitivity, excitation–contraction coupling, or ion transport processes. Additionally, a potential interaction with proton and lactate transport systems (e.g., monocarboxylate transporters) may contribute to alterations in acid–base regulation. However, these mechanisms remain speculative and should be explored in future studies.

One possibility is that acute β-alanine ingestion may influence acid–base regulation through indirect pathways, such as alterations in amino acid transport, changes in ionic balance, or modulation of muscle excitability. Additionally, β-alanine has been suggested to influence calcium sensitivity and excitation–contraction coupling, which could potentially modify contractile responses during high-intensity exercise [38,39].

Another plausible explanation is that β-alanine may interact with transporters involved in proton and lactate exchange, such as monocarboxylate transporters (MCTs), thereby facilitating the removal of metabolic by-products and reducing intracellular acidosis [40]. Although speculative, these mechanisms warrant further investigation.

The absence of similar findings in the BAH condition suggests that the response to acute β-alanine supplementation may not follow a linear dose–response pattern [16,18]. This observation is particularly relevant, as it challenges the assumption that larger doses necessarily lead to greater ergogenic effects. Several factors may explain this finding, including increased inter-individual variability, differences in absorption kinetics, or a potential saturation of buffering-related mechanisms. Moreover, the previous literature on chronic supplementation has also reported substantial variability in individual responses, supporting the notion that an optimal dosing range may exist [16,17]. Further research is required to better understand the dose–response relationship and the factors influencing individual variability.

The moderate and significant association between pH and performance observed in the placebo group (*r* = −0.40, *p* = 0.050, *r*^2^ = 0.16) reflects the well-established role of exercise-induced acidosis as a limiting factor in high-intensity exercise [3]. Although this relationship sits right at the threshold of statistical significance, the coefficient of determination reveals that systemic pH changes account for 16.1% of the variance in distance covered. In the context of sports performance, this magnitude of association may be considered physiologically relevant. This finding supports the validity of the experimental model and indicates that, in the absence of supplementation, performance is closely linked to systemic acid–base disturbances, confirming that our exercise protocol was sufficiently demanding to induce metabolic fatigue. Importantly, the absence of this relationship in the β-alanine conditions suggests that supplementation may alter the physiological determinants of performance, shifting the limiting factor from systemic acidosis toward better buffering capacity.

From an applied perspective, these findings suggest that acute β-alanine supplementation at moderate doses may influence performance-related responses by reducing mechanical demands rather than increasing maximal output. However, these implications should be interpreted cautiously, as further studies are needed to confirm their relevance in real-world settings.

Despite these findings, several limitations should be acknowledged. First, exercise efficiency was not directly assessed using metabolic measurements, preventing definitive interpretation of the observed alterations in mechanical work. Second, the study design does not allow for direct assessment of intracellular buffering capacity or muscle carnosine content. Finally, the correlational nature of the analyses precludes causal inference.

## 5. Conclusions

In conclusion, acute β-alanine supplementation did not improve performance outcomes during the 10’-TT. However, the BAM condition exhibited a large correlation between bicarbonate-related responses and reductions in mechanical work without impairing performance, pointing to a potential reduction in the mechanical cost of exercise. This effect may be related to extracellular buffering kinetics, particularly bicarbonate dynamics, although this interpretation should be made with caution. Additionally, the absence of effects in the greater-dose condition highlights the importance of dose-specific responses and warrants further investigation.

## Figures and Tables

**Table 1:** Group descriptive characteristics.

Group	Age (y)	Height (cm)	Body Mass (kg)	Muscle Mass (kg)	Body Fat (%)	VO_2_max (ml/kg/min)
**PLA**	38.1 ± 13.5	174.0 ± 5.7	71.6 ± 6.6	56.8 ± 4.5	16.3 ± 3.6	58.3 ± 7.3
**BAM**	34.7 ± 12.4	177.3 ± 7.1	71.9 ± 8.2	57.4 ± 5.4	15.7 ± 3.3	55.4 ± 7.2
**BAH**	36.4 ± 11.1	174.5 ± 5.5	76.0 ± 11.2	58.3 ± 5.8	18.5 ± 6.1	54.8 ± 8.7

y = years; cm = centimeters; kg = kilograms; VO_2_max = maximum oxygen uptake. All data are reported as mean ± standard deviation.

**Table 2:** Dietary intake characteristics of the participants during the acute supplementation phase.

Variable	PLA	BAM	BAH	*p*-Value
**Energy intake (kcal·day^−1^)**	2228.1 ± 749.8	2172.5 ± 580.3	2240.7 ± 733.7	0.734
**Protein (g·day^−1^)**	119.6 ± 35.0	118.5 ± 34.9	121.3 ± 35.3	0.589
**Protein (g·kg^−1^·day^−1^)**	1.7 ± 0.6	1.6 ± 0.6	1.7 ± 0.6	0.791
**Carbohydrate (g·day^−1^)**	235.1 ± 111.0	226.7 ± 75.4	237.4 ± 109.0	0.809
**Fat (g·day^−1^)**	85.8 ± 28.0	83.8 ± 24.1	85.5 ± 27.4	0.592
**Histidine (g·day^−1^)**	2.00 ± 1.36	2.10 ± 1.37	2.00 ± 1.34	0.298

kcal·day^-1^ = kilocalories per day; g·day^-1^ = grams per day; g·kg^−1^·day^−1^ = grams per kilogram per day. Data are presented as mean ± standard deviation (SD).

**Table 3:** Performance results from 10’-TT at control and following acute consumption of the product.

		Control	Acute Effects	*p*-Value
**Distance (Km)**	**PLA**	5.48 ± 0.47	5.53 ± 0.41	0.974
	**BAM**	5.38 ± 0.40	5.42 ± 0.44	
	**BAH**	5.54 ± 0.42	5.57 ± 0.44	
**Work (kJ)**	**PLA**	158.5 ± 31.7	160.3 ± 28.4	0.028
	**BAM**	154.0 ± 24.9	148.5 ± 26.1 *	
	**BAH**	161.2 ± 29.8	159.7 ± 28.5	
**MPO (W)**	**PLA**	263.5 ± 52.7	267.2 ± 47.5	0.038
	**BAM**	255.2 ± 41.6	247.5 ± 43.9 *	
	**BAH**	268.3 ± 49.7	266.1 ± 47.8	
**Relative Power (W·kg^−1^)**	**PLA**	3.51 ± 0.66	3.59 ± 0.69	0.046
	**BAM**	3.56 ± 0.56	3.47 ± 0.63 *	
	**BAH**	3.75 ± 0.63	3.73 ± 0.59	
**Average Speed (Km/h)**	**PLA**	32.8 ± 2.7	32.7 ± 3.6	0.854
	**BAM**	32.3 ± 2.4	32.5 ± 2.6	
	**BAH**	33.2 ± 2.5	33.4 ± 2.6	
**Cadence (RPM)**	**PLA**	93.3 ± 9.4	92.3 ± 10.6	0.654
	**BAM**	94.2 ± 9.6	94.0 ± 10.4	
	**BAH**	89.2 ± 7.8	87.9 ± 8.8	
**Heart Rate (BPM)**	**PLA**	165.6 ± 15.9	166.6 ± 11.1	0.914
	**BAM**	165.0 ± 21.7	164.4 ± 21.5	
	**BAH**	171.8 ± 17.0	170.8 ± 11.7	

Km = kilometers; kJ = kilojoules; MPO = mean power output; W = watts; W·kg^−1^ = watts per kilogram; Km/h = kilometers per hour; RPM = revolutions per minute; BPM = beats per minute * Significant differences (*p* < 0.050) were found between baseline measurements and those taken after acute consumption of the product.

**Table 4:** Ratings of Perceived Exertion (RPE) at different time points.

		Control	Acute Effects	*p*-Value
**Basal**	**PLA**	3.05 ± 2.01	2.59 ± 2.00	0.581
	**BAM**	2.46 ± 1.53	2.46 ± 1.79	
	**BAH**	2.63 ± 2.04	2.74 ± 1.81	
**Pre-10’-TT**	**PLA**	4.36 ± 1.33	4.18 ± 0.80	0.466
	**BAM**	4.62 ± 0.94	4.12 ± 1.07 *	
	**BAH**	4.63 ± 1.18	4.56 ± 1.42	
**Post-10’-TT**	**PLA**	9.09 ± 0.87	9.41 ± 0.73	0.304
	**BAM**	9.23 ± 0.86	9.15 ± 0.78	
	**BAH**	9.19 ± 0.68	9.15 ± 1.06	

* Significant differences were found between baseline measurements and those taken after acute consumption of the product (*p* < 0.050).

**Table 5:** The Relationship Between Performance and Biochemistry for each group.

		PLA	BAM	BAH
ΔWork vs. ΔpH_Post 10’-TT	***r***	−0.307	−0.077	−0.046
	***p*** **-value**	0.165	0.709	0.820
ΔWork vs. ΔHCO_3_^−^_Post 10’-TT	***r***	−0.308	−0.545	−0.088
	***p*** **-value**	0.163	0.004	0.664
ΔWorkPerKm vs. ΔpH_Post 10’-TT	***r***	−0.044	−0.052	0.085
	***p*** **-value**	0.847	0.800	0.672
ΔWorkPerKm vs. ΔHCO_3_^−^_Recovery	***r***	0.061	−0.282	−0.020
	***p*** **-value**	0.786	0.163	0.922
ΔDistance vs. ΔpH	***r***	−0.401	−0.081	−0.168
	***p*** **-value**	0.050	0.695	0.401

## Data Availability

Data are available from the corresponding author upon request.

## Supplementary Materials

The following supporting information can be downloaded at: https://www.mdpi.com/article/10.3390/sports14060252/s1, Figure S1: Flow diagram of participant recruitment and allocation; Table S1: Blood acid–base and metabolic responses.

## Abbreviations

The following abbreviations are used in this manuscript:

H+	Hydrogen ions
HCO_3_^−^	Bicarbonate
10’-TT	10- min time trieal
BAM	Moderate-dose β-alanine
BAH	High-dose β-alanine
RCTs	Randomized controlled trials
MPO	Mean power output
MIRT	Maximal incremental ramp test
VO_2_	Oxygen uptake
RPM	Revolutions per minute
Na+	Sodium
K+	Potassium
RPE	Rate of perceived exertion
WorkPerKm	Work relative to distance
VO_2_max	Maximum oxygen uptake
tCO_2_	Total CO_2_
SBE	Standard base excess
ΔWork	Changes in total mechanical work
ΔHCO_3_^−^	Changes in bicarbonate
