# Supplementary material for: Acute Moderate-Dose β-Alanine Improves Exercise Efficiency via Bicarbonate-Related Mechanisms During a Cycling Time Trial
Source: Sports (Basel). 2026 Jun 20;14(6):252. doi: 10.3390/sports14060252 (PMC13306792; doi:10.3390/sports14060252)
Supplement: Supplementary file 1 [file sports-14-00252-s001.zip › sports-4323801-supplementary.pdf]

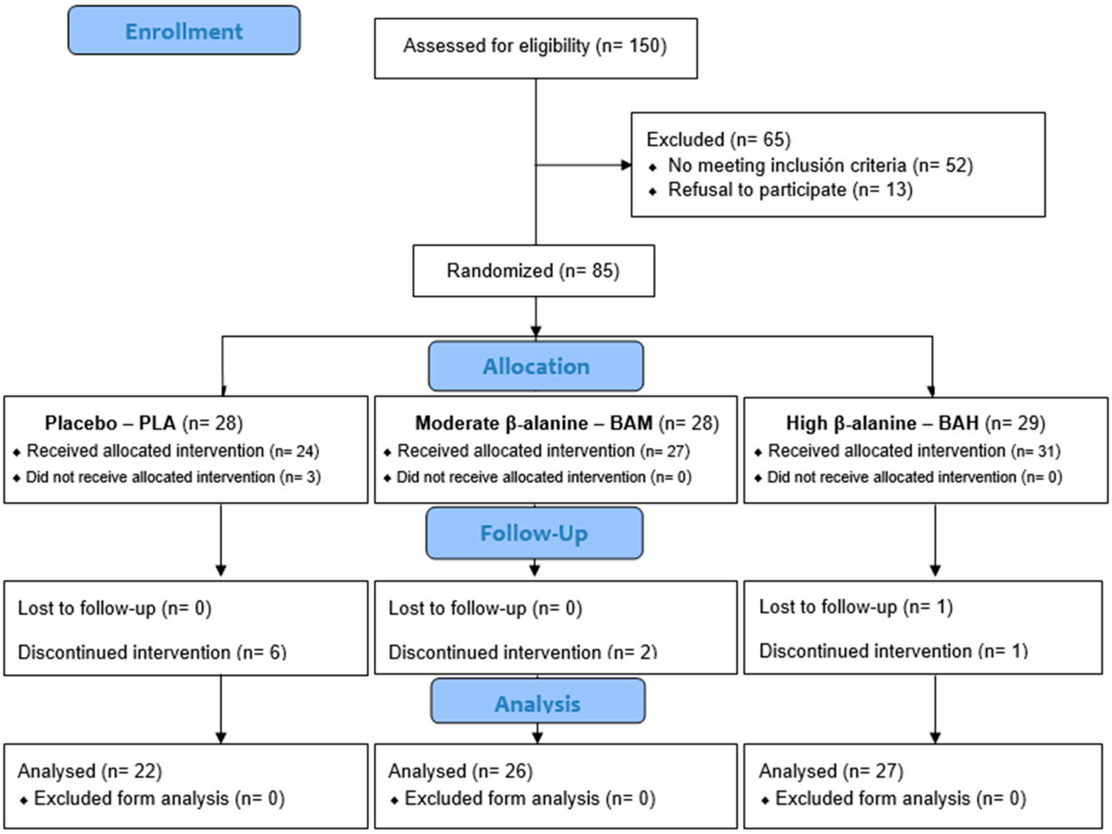

Figure S1. Flow diagram of participant recruitment and allocation

Table S1: Blood acid–base and metabolic responses.

		Basal		Pre – 10’ TT		Post – 10’ TT		Recovery	
		**Control**	**Acute Effects**	**Control**	**Acute Effects**	**Control**	**Acute Effects**	**Control**	**Acute Effects**
**pH**									
	**Placebo**	7.43 ± 0.04	7.42 ± 0.03	7.40 ± 0.04	7.40 ± 0.03	7.29 ± 0.05	7.28 ± 0.04	7.31 ± 0.05	7.29 ± 0.05
	**BAM**	7.44 ± 0.03	7.43 ± 0.03	7.39 ± 0.03	7.40 ± 0.03	7.28 ± 0.06	7.31 ± 0.07 *	7.29 ± 0.07	7.31 ± 0.08 *
	**BAH**	7.42 ± 0.02	7.42 ± 0.02	7.37 ± 0.04	7.39 ± 0.05 *	7.26 ± 0.06	7.28 ± 0.06	7.28 ± 0.06	7.29 ± 0.06
**HCO_3^-, mmol·L^{-1}**									
	**Placebo**	25.91 ± 1.33	25.90 ± 1.51	23.57 ± 2.36	23.48 ± 2.37	15.18 ± 3.35	14.77 ± 2.73	16.03 ± 3.30	15.33 ± 2.57
	**BAM**	26.50 ± 1.43	26.69 ± 1.65	23.45 ± 2.37	24.10 ± 2.30	15.03 ± 2.96	15.94 ± 2.97 *	15.29 ± 3.29	15.97 ± 3.41 *
	**BAH**	25.78 ± 1.35	25.81 ± 1.51	22.82 ± 3.06	22.80 ± 3.91	14.68 ± 2.60	15.62 ± 2.63 *	14.63 ± 2.89	15.39 ± 2.70 *
**CO_2, mmHg**									
	**Placebo**	39.22 ± 3.63	39.90 ± 2.33	37.86 ± 2.99	37.71 ± 2.73	30.75 ± 4.10	31.35 ± 3.85	31.55 ± 4.25	31.36 ± 3.22
	**BAM**	40.11 ± 3.20	39.88 ± 3.42	38.86 ± 2.87	38.84 ± 2.40	32.03 ± 3.53	32.83 ± 3.54	31.20 ± 3.27	31.86 ± 3.29
	**BAH**	39.63 ± 2.50	39.73 ± 2.39	38.81 ± 3.24	38.84 ± 3.44	31.49 ± 2.76	32.04 ± 3.25	30.83 ± 2.78	30.90 ± 2.18
**tCO_2, mmol·L^{-1}**									
	**Placebo**	27.12 ± 1.39	27.11 ± 1.54	24.73 ± 2.43	24.61 ± 2.41	16.13 ± 3.47	15.74 ± 2.85	17.00 ± 3.38	16.29 ± 2.64
	**BAM**	27.74 ± 1.49	27.92 ± 1.72	24.67 ± 2.43	25.29 ± 2.35 *	15.97 ± 3.04	16.91 ± 2.88 *	16.20 ± 3.36	16.95 ± 3.68 *
	**BAH**	27.00 ± 1.40	27.00 ± 1.55	24.02 ± 3.14	24.49 ± 3.08	15.50 ± 2.54	16.53 ± 2.47 *	15.49 ± 2.84	16.32 ± 2.43 *
**SBE, mmol·L^{-1}**									
	**Placebo**	1.60 ± 1.51	1.42 ± 1.81	-1.20 ± 2.77	-1.40 ± 2.75	-11.29 ± 4.04	-12.00 ± 3.33	-10.23 ± 3.92	-11.19 ± 3.19 *
	**BAM**	2.17 ± 1.50	2.45 ± 1.84	-1.51 ± 2.75	-0.69 ± 2.73 *	-11.87 ± 3.87	-10.82 ± 4.28 *	-11.35 ± 4.30	-10.75 ± 4.87
	**BAH**	1.34 ± 1.54	1.35 ± 1.70	-2.37 ± 3.66	-2.22 ± 4.68	-12.47 ± 3.38	-11.22 ± 3.26 *	-12.27 ± 3.61	-11.25 ± 3.35 *
**anionGAP, mmol·L^{-1}**									
	**Placebo**	10.23 ± 1.51	10.51 ± 2.11	13.68 ± 3.80	13.72 ± 3.59	21.66 ± 3.86	21.65 ± 3.06	20.90 ± 3.74	20.84 ± 3.16

	**BAM**	10.04 ± 1.71	10.20 ± 2.71	13.85 ± 6.25	14.12 ± 6.57	22.00 ± 3.49	22.28 ± 3.80	21.16 ± 3.59	21.68 ± 4.20
	**BAH**	9.87 ± 1.55	10.42 ± 1.37	13.33 ± 5.18	13.31 ± 4.45	22.93 ± 3.11	21.99 ± 3.03	22.20 ± 3.56	22.10 ± 3.16
**[La+]peak, mmol·L^{-1}**									
	**Placebo**	1.60 ± 0.38	1.51 ± 0.43	4.02 ± 2.28	3.47 ± 2.15	12.03 ± 2.91	11.93 ± 2.56	10.36 ± 2.82	10.19 ± 2.29
	**BAM**	1.55 ± 0.42	1.71 ± 0.44	3.78 ± 2.18	3.89 ± 1.90	12.49 ± 2.94	12.95 ± 3.77	10.84 ± 3.03	11.50 ± 3.89
	**BAH**	1.60 ± 0.44	1.64 ± 0.62	4.45 ± 2.92	4.20 ± 3.13	12.90 ± 2.71	13.44 ± 2.78	11.27 ± 2.74	12.06 ± 3.03 *

* Significant differences were found between baseline measurements and those taken after acute consumption of the product. HCO_3^-= Bicarbonate; tCO_2= total CO_2; SBE= excess standard base; [La+]peak, mmol·L^{-1}: peak lactate concentration; BAM= moderate-dose β-alanine; BAH= high-dose β-alanine
